# Supplementary material for: A Likelihood Approach for Real-Time Calibration of Stochastic Compartmental Epidemic Models
Source: PLoS Comput Biol. 2017 Jan 17;13(1):e1005257. doi: 10.1371/journal.pcbi.1005257 (PMC5240920; doi:10.1371/journal.pcbi.1005257)
Supplement: S1 File — (TAR.GZ) [file pcbi.1005257.s014.tar.gz › HSPH_Online-SI-Revision/Additions/MSS11/n100-extreme_noisy/n100-extreme_noisy_NEW_table-8.pdf]

1	R0	{2.76052, 16.3913, 0.1}	{2.10474, 8.71457, 0.01}	{{1.44762, 4.00571, 0.}, {2.64721, 12.5037, 0.}}	{24.4327, 46.8342, 108.}	inf	695.	545.767	{184.646, 827.376}	37.1325	0.260367	newly	289.367	727.72	{297., 1114.}	160.827	0.418622	Reff	2.73137	2.08156	{1.43669, 2.61361}	24.4172	ar	92 090.	76 436.6	{54 100., 91 011.}	17.1754
2	R0	{2.18202, 15.1734, 3.}	{2.55576, 15.2506, 0.02}	{{1.93539, 9.63533, 0.}, {2.96978, 18.8857, 0.}}	{26.2549, 26.7819, 99.3333}	inf	2011.	2326.02	{1436.23, 2979.44}	38.6833	0.174806	newly	1160.	1472.22	{1237., 1775.}	30.4862	0.116176	Reff	2.10076	2.45295	{1.86942, 2.83933}	25.4368	ar	84 264.	86 545.6	{77 853., 93 886.}	11.3586
3	R0	{2.57891, 12.5971, 1.}	{1.85496, 13.4392, 0.}	{{1.13227, 5.71232, 0.}, {2.52588, 19.3966, 0.}}	{29.7372, 29.2269, 100.}	inf	340.	37.8883	{1.41229, 107.97}	88.8564	1.40323	newly	303.	35.03	{0., 89.}	90.3267	1.56817	Reff	2.5648	1.85337	{1.13207, 2.5178}	29.4765	ar	90 302.	60 901.8	{13., 89 716.}	33.0104
4	R0	{2.5856, 9.28508, 0.1}	{2.01962, 12.0766, 0.}	{{1.22252, 5.83416, 0.}, {2.77394, 18.4504, 0.}}	{25.011, 46.9659, 100.}	inf	8.	6.09539	{0.877718, 14.1668}	75.3252	0.485967	newly	20.	12.4	{0., 26.}	102.4	0.972228	Reff	2.58508	2.01939	{1.22249, 2.77327}	25.006	ar	90 312.	57 623.8	{3., 92 092.}	36.9887
5	R0	{2.4406, 12.1341, 1.}	{1.88628, 13.6673, 0.}	{{1.14639, 6.0871, 0.}, {2.56757, 18.9086, 0.}}	{26.5601, 33.33, 100.}	inf	184.	21.8808	{1.14126, 57.3855}	88.1083	1.34844	newly	170.	23.91	{0., 51.}	88.3	1.55219	Reff	2.43279	1.88535	{1.14636, 2.56648}	26.4385	ar	88 597.	57 560.2	{6., 89 896.}	36.1065
6	R0	{2.72155, 15.4094, 2.}	{1.7214, 12.9321, 0.02}	{{1.08432, 6.0871, 0.}, {2.56757, 18.834, 0.}}	{37.5583, 25.5287, 99.}	inf	411.	52.1409	{1.10211, 168.261}	87.3136	1.51404	newly	302.	43.9	{0., 158.}	85.8477	1.57326	Reff	2.7028	1.71915	{1.08427, 2.56366}	37.2637	ar	91 682.	50 231.6	{10., 90 390.}	45.4236
7	R0	{2.63781, 14.3935, 0.1}	{1.78697, 14.0285, 0.}	{{1.12882, 8.26611, 0.}, {2.30907, 18.9086, 0.}}	{33.4646, 22.9663, 100.}	inf	448.	118.015	{1.6832, 297.658}	82.5993	1.22713	newly	307.	77.41	{1., 215.}	83.3388	1.32122	Reff	2.61787	1.78199	{1.12861, 2.29518}	33.1049	ar	91 192.	57 321.7	{15., 86 539.}	37.42
8	R0	{2.82391, 16.104, 1.}	{1.88108, 13.2503, 0.}	{{1.16434, 6.71734, 0.}, {2.52368, 18.834, 0.}}	{33.9071, 23.9676, 100.}	inf	560.	81.9645	{2.165, 183.568}	86.7471	1.37492	newly	385.	75.89	{1., 183.}	85.2182	1.42545	Reff	2.79954	1.87758	{1.16423, 2.5067}	33.5218	ar	92 664.	62 425.5	{16., 89 570.}	32.7661
9	R0	{2.80645, 14.6457, 0.1}	{1.82399, 13.867, 0.}	{{1.12882, 8.14942, 0.}, {2.59755, 18.834, 0.}}	{35.6303, 23.3799, 100.}	inf	226.	49.2661	{1.58904, 156.651}	80.3572	1.17518	newly	148.	36.58	{1., 104.}	82.5811	1.24533	Reff	2.79573	1.82193	{1.12873, 2.59441}	35.4757	ar	92 567.	57 769.6	{8., 90 333.}	37.7472
10	R0	{1.86213, 5.4271, 0.1}	{1.95952, 12.1665, 0.}	{{1.13754, 6.00596, 0.}, {2.66225, 18.2464, 0.}}	{24.1315, 126.931, 100.}	inf	32.	11.2237	{1.01563, 30.153}	75.9812	0.794135	newly	74.	15.2	{0., 41.}	85.2973	1.30163	Reff	1.8607	1.95907	{1.13748, 2.66203}	24.1426	ar	75 362.	65 568.1	{5., 91 028.}	28.8153
11	R0	{2.27349, 14.6338, 1.}	{2.37311, 12.5282, 0.02}	{{1.93419, 8.56054, 0.}, {2.7499, 15.6124, 0.}}	{13.4555, 20.8604, 98.}	inf	1278.	985.824	{657.918, 1326.33}	27.8204	0.163638	newly	843.	831.15	{448., 1181.}	22.7129	0.106917	Reff	2.22036	2.33141	{1.89668, 2.69583}	13.6322	ar	85 805.	85 386.2	{77 778., 92 232.}	6.72597
12	R0	{2.77261, 14.1894, 0.1}	{1.70064, 13.9155, 0.02}	{{1.10202, 8.26611, 0.}, {2.42205, 19.4475, 0.}}	{38.9794, 26.605, 116.}	inf	206.	47.949	{1.15661, 164.524}	81.2667	1.18673	newly	178.	32.07	{0., 93.}	83.9494	1.3856	Reff	2.76385	1.69862	{1.10178, 2.4193}	38.8481	ar	92 508.	52 481.4	{9., 88 096.}	43.3275
13	R0	{2.65402, 19.3935, 2.}	{2.3072, 13.6097, 0.}	{{1.28596, 3.98513, 0.}, {2.96797, 18.8857, 0.}}	{18.8912, 30.0662, 100.}	inf	1354.	822.852	{170.013, 1503.62}	44.1529	0.345361	newly	658.	574.02	{254., 915.}	33.1094	0.191815	Reff	2.59651	2.2716	{1.27709, 2.89954}	18.4863	ar	91 184.	80 166.6	{40 080., 93 613.}	13.5169
14	R0	{1.75297, 4.45354, 0.1}	{1.8259, 13.1961, 0.01}	{{1.14344, 6.71734, 0.}, {2.6365, 18.4042, 0.}}	{28.3494, 199.768, 108.}	inf	18.	23.2693	{1.69892, 58.3556}	116.366	0.540314	newly	29.	22.39	{0., 53.}	100.655	0.895449	Reff	1.75187	1.82495	{1.14337, 2.63475}	28.3328	ar	71 263.	57 144.8	{7., 91 148.}	38.8915
15	R0	{1.99928, 7.1501, 0.1}	{1.8162, 12.8399, 0.}	{{1.13726, 5.43578, 0.}, {2.59755, 18.4042, 0.}}	{25.359, 89.5568, 100.}	inf	97.	20.1697	{1.1842, 57.3396}	81.9731	1.12423	newly	168.	24.22	{1., 39.}	89.2738	1.53408	Reff	1.99548	1.81532	{1.13714, 2.59714}	25.3584	ar	79 602.	55 053.7	{10., 90 320.}	38.1716
16	R0	{2.67398, 10.3134, 1.}	{1.91653, 13.7856, 0.}	{{1.13726, 8.31782, 0.}, {2.74056, 19.1478, 0.}}	{29.8766, 44.4796, 100.}	inf	44.	29.0901	{1.90467, 61.2917}	87.1182	0.715824	newly	54.	30.69	{1., 49.}	100.463	0.98985	Reff	2.67219	1.91532	{1.13707, 2.72943}	29.8633	ar	91 289.	59 155.9	{9., 91 892.}	35.6069
17	R0	{2.44634, 16.3226, 0.1}	{2.51109, 15.0326, 0.}	{{1.83592, 9.63533, 0.}, {2.96978, 18.8857, 0.}}	{17.9432, 21.655, 100.}	inf	1288.	1085.67	{245.231, 1668.49}	31.566	0.20498	newly	777.	706.56	{385., 981.}	23.5393	0.125417	Reff	2.39443	2.46343	{1.82898, 2.90404}	17.8143	ar	88 710.	85 001.9	{75 067., 93 840.}	9.93303
18	R0	{2.02168, 10.1676, 0.1}	{1.85054, 13.9904, 0.01}	{{1.10202, 8.35044, 0.}, {2.6365, 19.3966, 0.}}	{25.5228, 46.4426, 108.}	inf	354.	148.04	{2.36615, 434.867}	75.1387	1.00488	newly	351.	95.09	{1., 254.}	76.208	1.19582	Reff	2.00733	1.84425	{1.10173, 2.60202}	25.3659	ar	80 299.	60 659.	{18., 90 928.}	31.9117
19	R0	{2.89969, 15.009, 0.1}	{1.84493, 13.8842, 0.}	{{1.13754, 8.26611, 0.}, {2.59755, 19.3966, 0.}}	{36.4497, 20.1442, 100.}	inf	224.	49.8654	{2.06833, 117.767}	80.2933	1.09171	newly	179.	35.9	{1., 97.}	82.8045	1.20983	Reff	2.88972	1.84287	{1.13743, 2.59447}	36.3053	ar	93 349.	61 080.	{13., 90 726.}	34.5818
20	R0	{2.68933, 7.34928, 0.1}	{1.98627, 12.0056, 0.02}	{{1.18979, 5.43578, 0.}, {2.8702, 18.9003, 0.}}	{28.7537, 75.4659, 116.}	inf	3.	8.55944	{1.01508, 13.4148}	224.043	0.401348	newly	7.	25.84	{0., 29.}	369.143	0.752826	Reff	2.68909	1.98593	{1.18976, 2.86863}	28.7515	ar	91 574.	54 237.4	{4., 92 647.}	41.3161
21	R0	{2.15352, 9.28074, 0.1}	{1.78786, 13.545, 0.}	{{1.08432, 6.00596, 0.}, {2.59755, 18.834, 0.}}	{28.0588, 57.3617, 100.}	inf	131.	22.9004	{0.8416, 65.4486}	84.0941	1.24152	newly	151.	24.3	{0., 62.}	87.6556	1.56623	Reff	2.14827	1.78693	{1.08428, 2.59679}	28.0548	ar	83 845.	51 462.3	{5., 90 594.}	42.6709
22	R0	{2.21953, 9.04387, 0.1}	{1.87051, 12.7023, 0.}	{{1.14639, 5.15387, 0.}, {2.73108, 18.8857, 0.}}	{25.5209, 58.4927, 100.}	inf	114.	23.407	{1.38522, 47.5867}	87.7146	1.19154	newly	136.	35.93	{0., 101.}	92.625	1.45627	Reff	2.21565	1.86954	{1.14634, 2.72989}	25.5005	ar	84 856.	58 195.6	{7., 91 652.}	34.8791
23	R0	{2.21987, 14.5347, 1.}	{2.36828, 14.6724, 0.}	{{1.63746, 10.1332, 0.}, {2.93978, 19.9817, 0.}}	{17.2619, 22.6498, 100.}	inf	1324.	1216.49	{396.323, 1709.4}	27.1831	0.160364	newly	822.	801.55	{532., 1026.}	17.5754	0.0863152	Reff	2.16759	2.31563	{1.63157, 2.86457}	17.0259	ar	85 067.	83 401.1	{66 216., 93 629.}	9.99232
24	R0	{2.84268, 17.9659, 1.}	{1.68047, 13.2457, 0.}	{{1.13726, 8.35044, 0.}, {2.24242, 19.4475, 0.}}	{40.8844, 28.8303, 100.}	inf	799.	148.513	{1.87521, 382.279}	81.4126	1.28805	newly	439.	96.13	{1., 266.}	78.1025	1.22215	Reff	2.80769	1.67432	{1.1368, 2.22563}	40.3667	ar	92 836.	56 314.5	{38., 85 329.}	39.3398
25	R0	{2.52706, 10.3679, 0.1}	{1.69742, 13.4705, 0.01}	{{1.10202, 6.00596, 0.}, {2.37462, 19.4475, 0.}}	{33.5475, 46.8249, 108.}	inf	97.	39.9197	{1.88513, 81.9376}	74.6259	0.860306	newly	97.	28.32	{1., 65.}	78.2887	1.04772	Reff	2.52279	1.69573	{1.10178, 2.37234}	33.488	ar	89 623.	53 484.3	{13., 87 474.}	40.5326
26	R0	{2.62835, 19.1088, 1.}	{2.44426, 14.2456, 0.}	{{1.93539, 9.63533, 0.}, {2.96978, 18.8857, 0.}}	{15.3211, 25.5413, 100.}	inf	1699.	1155.78	{637.922, 1677.52}	32.2219	0.223179	newly	814.	803.27	{457., 1085.}	22.4877	0.109223	Reff	2.55757	2.39434	{1.89985, 2.90351}	15.1499	ar	90 908.	84 827.1	{77 357., 93 788.}	8.75958
27	R0	{2.76831, 19.4155, 2.}	{2.43774, 12.2066, 0.}	{{1.93419, 8.56054, 0.}, {2.96947, 15.963, 0.}}	{14.9933, 37.1294, 100.}	inf	2186.	1593.34	{941.899, 2228.66}	28.3579	0.164637	newly	1084.	1363.47	{906., 1639.}	31.2943	0.117395	Reff	2.67358	2.37006	{1.89682, 2.86879}	14.4998	ar	92 175.	87 197.9	{77 882., 93 874.}	6.15792
28	R0	{2.96609, 16.8903, 1.}	{1.88577, 12.2691, 0.}	{{1.13754, 6.00596, 0.}, {2.66225, 18.9003, 0.}}	{36.44, 31.762, 100.}	inf	591.	68.5588	{1.73425, 260.175}	89.0297	1.58869	newly	383.	90.59	{1., 411.}	86.8903	1.5629	Reff	2.94132	1.88286	{1.13747, 2.66164}	36.0295	ar	94 024.	61 512.8	{19., 91 223.}	34.5794
29	R0	{2.17046, 14.39, 0.1}	{2.37622, 14.8494, 0.02}	{{1.81647, 10.5184, 0.}, {2.93978, 19.9817, 0.}}	{18.244, 22.1307, 116.}	inf	1169.	1201.9	{377.998, 1754.51}	32.8023	0.168526	newly	743.	766.15	{491., 930.}	20.747	0.0957056	Reff	2.12193	2.32468	{1.80011, 2.86251}	17.8941	ar	84 001.	84 030.8	{74 047., 93 659.}	9.91538
30	R0	{2.88772, 12.4647, 1.}	{2.04243, 12.7634, 0.01}	{{1.35081, 5.58477, 0.}, {2.9194, 19.1478, 0.}}	{29.8958, 34.1378, 99.}	inf	43.	11.8557	{0.84186, 15.5387}	93.189	1.07962	newly	59.	64.51	{0., 32.}	174.898	1.45127	Reff	2.88584	2.04196	{1.35078, 2.9184}	29.8751	ar	93 273.	59 819.7	{2., 93 483.}	35.9905
31	R0	{2.46831, 8.14308, 0.1}	{2.00737, 11.1054, 0.}	{{1.18979, 4.06661, 0.}, {2.86001, 18.4042, 0.}}	{25.1483, 61.0325, 100.}	inf	13.	5.59235	{0.532406, 8.68362}	87.1903	0.792766	newly	16.	49.81	{0., 19.}	350.438	1.1001	Reff	2.46792	2.00718	{1.18977, 2.85979}	25.1453	ar	88 762.	55 937.2	{2., 92 251.}	38.788
32	R0	{1.93236, 7.38987, 0.1}	{1.9182, 12.4591, 0.}	{{1.14344, 6.0871, 0.}, {2.7499, 18.834, 0.}}	{25.8545, 76.1489, 100.}	inf	96.	25.3891	{1.07793, 66.425}	85.3842	1.09077	newly	158.	41.37	{0., 95.}	94.4367	1.481	Reff	1.92828	1.91715	{1.14338, 2.74906}	25.8621	ar	77 654.	60 352.	{7., 92 173.}	33.7814
33	R0	{2.82251, 17.6954, 1.}	{1.88071, 12.837, 0.01}	{{1.14639, 6.00596, 0.}, {2.52588, 18.8857, 0.}}	{34.0341, 30.0805, 99.}	inf	586.	60.2191	{1.66988, 171.701}	90.09	1.50281	newly	325.	60.42	{1., 160.}	86.2092	1.43087	Reff	2.7977	1.87821	{1.14633, 2.51769}	33.6332	ar	92 745.	61 583.3	{14., 90 115.}	33.7524
34	R0	{1.87083, 12.3994, 1.}	{2.43689, 14.7063, 0.01}	{{1.3618, 4.98002, 0.}, {2.88523, 18.2812, 0.}}	{36.8854, 37.0998, 99.}	inf	1336.	1682.85	{424.305, 2732.03}	43.4037	0.182288	newly	975.	1094.08	{804., 1471.}	23.5918	0.0933803	Reff	1.81905	2.36337	{1.33908, 2.76728}	36.3956	ar	76 282.	83 880.7	{47 612., 93 457.}	20.3683
35	R0	{2.95779, 16.3003, 1.}	{1.84951, 14.4952, 0.}	{{1.14344, 8.26611, 0.}, {2.59755, 19.4475, 0.}}	{37.5172, 22.9478, 100.}	inf	233.	27.3262	{1.49794, 90.3747}	89.2032	1.37543	newly	159.	20.15	{0., 54.}	88.8616	1.48228	Reff	2.94682	1.84833	{1.14328, 2.59563}	37.3501	ar	93 873.	59 627.3	{16., 90 234.}	36.4823
36	R0	{2.18718, 14.261, 3.}	{2.32334, 11.0025, 0.}	{{1.24751, 2.5364, 0.}, {2.96947, 15.963, 0.}}	{18.9451, 26.3383, 100.}	inf	1258.	805.716	{265.659, 1120.71}	38.5658	0.25934	newly	857.	811.28	{474., 1153.}	23.8786	0.115842	Reff	2.13862	2.2888	{1.23448, 2.9234}	19.3149	ar	84 379.	81 806.6	{37 990., 93 704.}	12.3841
37	R0	{2.44912, 9.31118, 0.1}	{1.98974, 12.572, 0.}	{{1.14344, 4.56746, 0.}, {2.88523, 18.834, 0.}}	{25.8535, 53.8476, 100.}	inf	41.	16.5959	{1.2099, 29.3689}	91.2708	0.914038	newly	54.	38.18	{0., 73.}	126.704	1.25543	Reff	2.44728	1.98906	{1.1434, 2.88467}	25.832	ar	88 442.	59 805.2	{4., 93 178.}	34.6161
38	R0	{2.05949, 5.72514, 0.1}	{1.86263, 11.5114, 0.01}	{{1.14639, 5.15387, 0.}, {2.64721, 17.1868, 0.}}	{25.6598, 107.994, 108.}	inf	11.	10.1413	{0.950499, 24.4121}	85.3775	0.536085	newly	26.	16.44	{0., 49.}	99.6154	0.965524	Reff	2.05842	1.86223	{1.14637, 2.64645}	25.6586	ar	81 587.	53 697.6	{4., 90 897.}	40.498
39	R0	{1.75505, 8.52404, 0.1}	{2.1995, 13.1663, 0.01}	{{1.25474, 8.35044, 0.}, {2.88523, 18.2812, 0.}}	{33.8778, 64.2304, 108.}	inf	489.	489.963	{27.0498, 935.725}	60.8179	0.438294	newly	574.	378.87	{22., 777.}	47.615	0.457007	Reff	1.73539	2.17867	{1.24671, 2.85601}	33.8691	ar	70 925.	77 590.3	{38 894., 93 286.}	26.0306
40	R0	{2.24724, 13.7861, 1.}	{2.29849, 10.626, 0.}	{{1.44762, 4.00571, 0.}, {2.53075, 12.5037, 0.}}	{15.4927, 25.3546, 100.}	inf	1281.	920.312	{425.898, 1500.32}	35.2591	0.226949	newly	906.	963.13	{599., 1449.}	31.4316	0.137007	Reff	2.19627	2.25947	{1.42408, 2.4854}	15.7288	ar	85 257.	82 195.4	{54 324., 89 929.}	10.4919
41	R0	{1.77635, 8.62406, 0.1}	{2.03229, 8.16868, 0.}	{{1.44762, 3.83841, 0.}, {2.64721, 12.5037, 0.}}	{27.5286, 38.1513, 100.}	inf	634.	514.012	{164.31, 817.859}	34.6464	0.233181	newly	682.	743.18	{381., 1071.}	31.0557	0.163853	Reff	1.75212	2.01048	{1.43632, 2.61413}	27.562	ar	72 941.	74 238.	{54 376., 91 114.}	20.9814
42	R0	{1.80397, 5.4932, 1.}	{1.90564, 11.9587, 0.01}	{{1.14344, 5.30671, 0.}, {2.80387, 18.2464, 0.}}	{28.1088, 123.129, 99.}	inf	36.	12.7319	{1.0209, 29.9729}	77.5963	0.855544	newly	83.	22.95	{0., 48.}	90.7108	1.39916	Reff	1.80242	1.90513	{1.14338, 2.80233}	28.122	ar	73 232.	58 056.6	{6., 92 385.}	38.7749
43	R0	{2.83096, 16.7077, 0.1}	{1.71653, 14.1876, 0.}	{{1.06738, 8.71343, 0.}, {2.27582, 19.1478, 0.}}	{39.3657, 21.5815, 100.}	inf	491.	108.251	{1.6858, 303.391}	80.2447	1.21828	newly	311.	69.62	{1., 184.}	80.5916	1.24024	Reff	2.80905	1.71208	{1.06604, 2.2713}	39.0513	ar	92 740.	55 971.9	{16., 85 778.}	39.6465
44	R0	{2.73678, 11.6021, 1.}	{1.82142, 12.2147, 0.01}	{{1.13726, 5.58477, 0.}, {2.66225, 18.4504, 0.}}	{34.355, 32.3428, 99.}	inf	128.	17.1135	{1.03726, 36.9503}	89.3399	1.31979	newly	125.	22.69	{0., 52.}	89.464	1.48882	Reff	2.73147	1.82069	{1.13718, 2.66173}	34.2648	ar	92 003.	53 651.7	{6., 91 402.}	41.8879
45	R0	{2.14235, 6.98601, 0.1}	{1.83657, 13.5465, 0.}	{{1.13754, 6.00596, 0.}, {2.66225, 19.1478, 0.}}	{26.2698, 102.405, 100.}	inf	49.	23.2562	{0.891483, 65.5266}	82.7743	0.863686	newly	74.	27.	{0., 84.}	93.	1.22601	Reff	2.14079	1.8356	{1.13746, 2.66119}	26.249	ar	82 974.	56 072.	{6., 91 107.}	37.1401
46	R0	{2.23409, 9.21289, 0.1}	{1.90871, 13.189, 0.01}	{{1.13726, 8.71343, 0.}, {2.73108, 18.8857, 0.}}	{23.2824, 49.6087, 108.}	inf	58.	28.6583	{1.63307, 55.9182}	84.3523	0.792337	newly	78.	28.99	{1., 46.}	93.3462	1.08498	Reff	2.23183	1.90753	{1.13712, 2.72618}	23.2405	ar	85 163.	62 099.5	{6., 91 797.}	30.0703
47	R0	{2.12258, 8.15265, 0.1}	{1.95155, 12.2522, 0.}	{{1.16434, 6.00596, 0.}, {2.81891, 18.4504, 0.}}	{25.2704, 59.9081, 100.}	inf	73.	11.8074	{0.686535, 30.3154}	84.0771	1.16731	newly	100.	16.71	{0., 53.}	86.01	1.41978	Reff	2.11978	1.95108	{1.16431, 2.81879}	25.2705	ar	82 690.	56 898.7	{4., 92 631.}	37.7491
48	R0	{2.71771, 10.4636, 0.1}	{1.93108, 12.9965, 0.}	{{1.14639, 6.25659, 0.}, {2.7499, 18.4042, 0.}}	{30.1046, 40.8877, 100.}	inf	20.	9.89638	{1.02696, 24.0593}	79.4911	0.711405	newly	23.	15.64	{0., 34.}	112.087	1.07395	Reff	2.71654	1.93069	{1.14636, 2.74954}	30.0911	ar	91 716.	60 057.6	{6., 92 039.}	34.8224
49	R0	{2.44369, 6.66477, 0.1}	{1.95286, 11.5229, 0.}	{{1.13726, 4.56746, 0.}, {2.7499, 17.2871, 0.}}	{26.7988, 86.798, 100.}	inf	7.	4.73254	{0.485844, 8.00123}	81.1451	0.582116	newly	14.	56.1	{0., 15.}	445.286	1.11543	Reff	2.44335	1.9527	{1.13723, 2.74985}	26.7957	ar	88 827.	53 073.8	{1., 92 016.}	41.7981
50	R0	{2.77383, 17.9775, 0.1}	{2.43359, 14.5646, 0.01}	{{1.67732, 9.44073, 0.}, {2.96978, 18.8857, 0.}}	{16.3868, 21.4634, 108.}	inf	1401.	982.948	{210.32, 1616.47}	36.9322	0.261838	newly	799.	655.67	{332., 964.}	28.9049	0.167757	Reff	2.71353	2.39077	{1.67085, 2.90369}	16.0155	ar	92 334.	84 120.8	{68 314., 93 842.}	9.82773
